# Inhibitory effect of IL-8 on insulin action in human adipocytes via MAP kinase pathway

**DOI:** 10.1186/1476-9255-6-25

**Published:** 2009-08-27

**Authors:** Chikaaki Kobashi, Sachie Asamizu, Manabu Ishiki, Minoru Iwata, Isao Usui, Katusuya Yamazaki, Kazuyuki Tobe, Masashi Kobayashi, Masaharu Urakaze

**Affiliations:** 1 First Department of Internal Medicine, Faculty of Medicine, Toyama University, Toyama, Japan

## Abstract

**Background:**

Various cytokines and other compounds are produced in human adipose tissue and might have functions in the adipose tissue. They might be involved in complications associated with obesity and diabetes. Recently, interleukin-8 (IL-8) has been shown to be produced and released from human adipose tissue and/or adipocytes, suggesting IL-8 involvement in some obesity-related health complications. Therefore, we found it of interest to investigate whether IL-8 is involved in the insulin action in human adipocytes.

**Methods:**

The IL-8 levels in the medium were measured using ELISA. The IL-8 mRNA expression was analyzed using Northern blot analysis. The phosphorylation of Akt was analyzed using Western blot analysis. Furthermore, we examined the effect of IL-8 on the phosphorylation of Akt induced by insulin.

**Results:**

The level of IL-8 in the medium and the IL-8 mRNA expression after stimulation with either TNF-α, IL-1β, or CRP was significantly enhanced in human adipocytes. It is particularly interesting that IL-8 per se also enhanced IL-8 mRNA expression. The IL-8 induced-IL-8 mRNA expression was inhibited by PD98059 (a MEK inhibitor) or SB203580 (a p38 MAPK inhibitor). The IL-8 inhibited insulin-induced Akt phosphorylation. The inhibitory effect of IL-8 was eliminated by either PD 98059 or SB203580.

**Conclusion:**

These data suggest that IL-8 is a main adipocytokine producing insulin resistance via the inhibition of insulin-induced Akt phosphorylation in adipocytes. The attenuation of IL-8 action might be a target for prevention of diabetes and its complications.

## Background

Insulin resistance is defined as the impaired ability of target tissues of fat, liver, and muscle to show various metabolic effects of insulin, including glucose uptake [1]. It is probably caused by defects in the insulin signal transduction pathways [2,3]. Insulin resistance is central to the pathophysiology of metabolic syndrome because it is associated with type 2 diabetes, hypertension, and dyslipidemia. In that state, the risk for cardiovascular diseases is increased [4]. Although the molecular mechanisms leading to development of insulin resistance are not fully understood, an association appears to exist between insulin resistance and the accumulation of abdominal visceral fat.

Adipocytes synthesize and secrete a variety of bioactive proteins in addition to their role in fat storage. During the development of obesity and type 2 diabetes these cells increase in size and number and their metabolic activity is dramatically altered. It is conceivable that some adipocyte-derived factors underlie the association of insulin resistance and increased risk for coronary heart disease [5]. Tumor necrosis factor-α (TNF-α) is also elevated in obesity and may contribute to many aspects of adipose tissue biology including development of insulin resistance and abnormalities in lipid metabolism. Hotamisligil et al. have reported that TNF-α inhibits the phosphorylation of Akt by insulin [6]. Insulin action is the consequence of insulin binding to its plasma membrane receptor and is transmitted through the cell by a series of protein-protein interactions such as insulin receptor substrates 1 and 2 (IRS-1 and IRS-2), phosphatidyl- inositol-3 (PI3)-kinase, and protein kinase (PK) B/Akt. Activation of PKB/Akt is a key step for initiating several of insulin's metabolic effects, including glucose uptake and GLUT-4 translocation [7,8]. Hill et al. reported that microinjection of a PKB/Akt substrate peptide or an antibody to PKB/Akt inhibited the effect of insulin-stimulated GLUT-4 translocation in adipocytes [9]. Therefore, it is conceivable that inhibition of insulin-induced Akt activation is reflected in insulin resistant states. Hauner et al. also have reported that TNF-α down-regulates the insulin-sensitive glucose transporter GLUT-4, consequently decreasing glucose uptake in adipocytes [10].

Recently, it was reported that TNF-α stimulates the IL-8 production in human adipocytes [11]. In fact, IL-8, a chemokines, is known to activate neutrophils. However, little evidence is available to describe the role of IL-8 in adipocytes, obesity, and the development of insulin resistance.

In the present study, we show that the IL-8 production in human adipocytes is enhanced by inflammatory substances such as TNF-α, IL-1β, and CRP, and we examined the effect of IL-8 on insulin-induced Akt phosphorylation in adipocytes.

## Methods

### Cell culture

Human adipocyte culture. Cryopreserved human subcutaneous preadipocytes derived from human adipose tissue were obtained (together with culture media) from Cambrex Bio Science, Walkersville, Inc. The cells were cultured with preadipocyte growth medium (PGM) containing 10% FCS, 2 mM L-glutamine, 100 units/ml penicillin and 100 μg/ml streptomycin to reach confluence. Then, cells were differentiated into mature adipocytes by incubation with adipocyte differentiation medium containing 10 μg/ml insulin, 1 μM dexamethasone, 200 μM indomethacin and 500 μM isobutylmethylxanthine.

### Measurement of IL-8 in the media

Mature adipocytes were stimulated using either TNF-α, IL-1β, or CRP for 18 h. For measurement of IL-8 release from adipocytes in the medium, the media were collected. Then the concentrations of IL-8 in the media were measured by enzyme linked immunosorbent assay (ELISA) [12].

### Northern blot analysis

Northern blot analysis was performed according to the method described previously [13]. Briefly, mature adipocytes were stimulated by either TNF-α, IL-1β, CRP, or IL-8 for 18 h. Then RNA from the adipocytes was extracted using ISOGEN (Nippon Gene, Japan). A digoxigenin-labeled probe for human IL-8 cDNA with dUTP by the random priming method (Roche Diagnostics Co.) was used for hybridization. The intensity of the bands was analyzed using NIH image.

### Western blot analysis

The phosphorylation of Akt (Thr 308) was analysed by a non-radioactive method using a commercial kit (New England Biolabs Inc. and Cell Signaling). Briefly, after pretreatment of mature adipocytes with IL-8 for the indicated period, cells were stimulated with insulin for 10 min. Then, cell lysates were prepared using lysis buffer. The cell lysates were loaded on sodium dodecyl sulphate (SDS)-polyacrylamide gel electrophoresis (PAGE), transferred onto a membrane (Millipore). The following antibodies were used: phospho-specific Akt (Thr308) antibody, Akt antibody (Cell Signaling), and an anti-rabbit secondary antibody conjugated to horseradish peroxidase (Amersham). The intensity of the bands was analysed by NIH image.

### Data analysis

Data are presented as the mean ± s.d. Statistical analyses were performed using ANOVA, followed by Scheffe's *t*-test. A value of *P *< 0.05 was considered significant.

## Results

**Figure 1:**
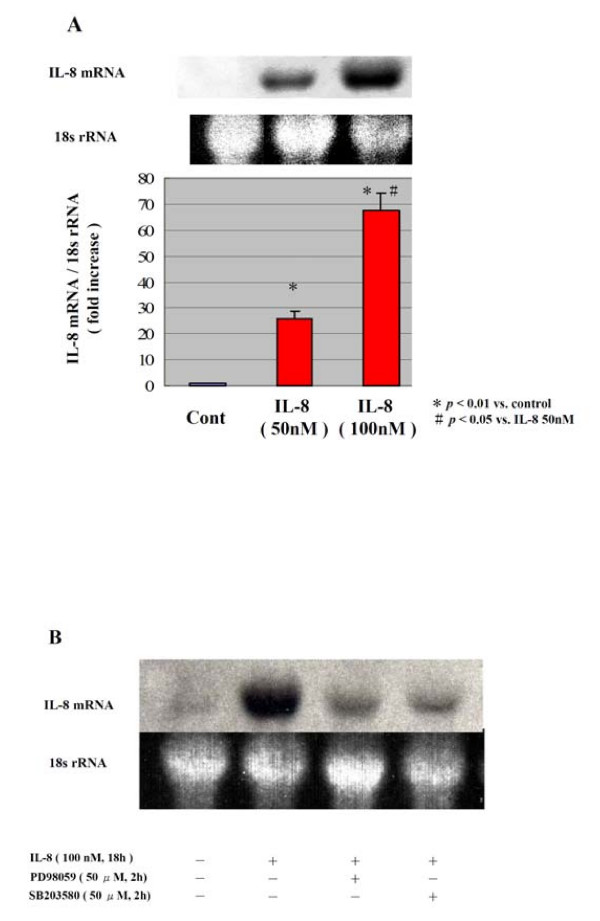
**IL-8 mRNA expression induced by IL-8 in human adipocytes**. Human adipocytes were stimulated with IL-8 (50 nM or 100 nM) for 18 h (A). The IL-8 mRNA expression was analyzed by Northern blot. For the experiments with inhibitors (B), 0.1% DMSO was added as vehicle control, and PD98059 (50 μM) or SB203580 (50 μM) was added 2 h before stimulation with IL-8 (100 nM). The intensity of the IL-8 mRNA band was corrected with that of the 18s rRNA band. The data are representative of three different experiments (Means ± SD). * *p *< 0.01 vs. control # *p *< 0.05 vs. IL-8 50 nM.

First, we examined the IL-8 release in the media using TNF-α, IL-1β, or CRP in human adipocytes. The IL-8 release in the media was increased in a time-dependent manner in all stimulators, although it was less by CRP than by TNF-α and IL-1β (data not shown). The IL-8 mRNA expression was clearly enhanced by either TNF-α, IL-1β, or CRP (data not shown). It is particularly interesting that, as presented in Fig. 1A, IL-8 per se also enhanced the IL-8 mRNA expression in human adipocytes. Those data suggest that inflammatory substances act on adipocytes to stimulate IL-8 production in humans. Fig. 1B shows that the IL-8 mRNA expression in human adipocytes by IL-8 was inhibited by PD98059 (a MEK inhibitor) or SB203580 (a p38 MAPK inhibitor). The IL-8 per se also activated the phosphorylation of ERK and p38 MAPK (data not shown).

**Figure 2:**
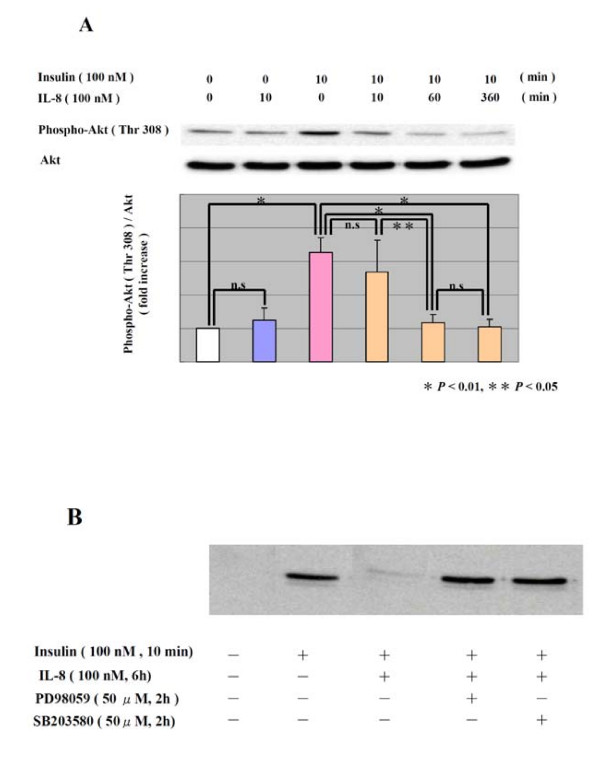
**Effect of IL-8 on phosphorylation of Akt in human adipocytes**. After treatment with IL-8 (100 nM) for the indicated period (A), human adipocytes were stimulated with insulin (100 nM) for 10 min. The phosphorylation of Akt induced by insulin (100 nM) was analyzed by Western blot. For the experiments with inhibitors (B), 0.1% DMSO was added as vehicle control, and PD98059 (50 μM) or SB203580 (50 μM) was added 2 h before stimulation with insulin (100 nM). The data are representative of three different experiments (Means ± SD). * *P *< 0.01, ** *P *< 0.05

Next, we examined the effect of IL-8 on the action of insulin in human adipocytes. Fig. 2A shows that the phosphorylation of Akt by insulin was diminished through pretreatment with IL-8 in a time-dependent manner. Fig. 2B shows that the inhibitory effect of IL-8 on Akt phosphorylation by insulin was eliminated by either PD 98059 or SB203580. Those data suggest that IL-8 inhibits the insulin signal pathway via the ERK pathway and p38 MAP kinase pathway.

## Discussion

In the present study, we demonstrate for the first time that IL-8 per se enhanced the IL-8 mRNA expression in human adipocytes and show that IL-8 has an inhibitory effect on the Akt phosphorylation induced by insulin.

Actually, IL-8 is a monomeric polypeptide and a well-studied member of the CXC chemokine family, which plays a crucial role in the recruitment of neutrophils and lymphocytes into tissues [14]. In fact, IL-8 is produced by a variety of cells including human adipocytes [11], frequently in response to inflammatory stimuli such as IL-1, or TNF-α [14]. Here, we also observed that TNF-α, IL-1β, and CRP enhanced IL-8 production in human adipocytes. It is particularly interesting that IL-8 per se also enhanced the IL-8 mRNA expression in human adipocytes. Recently, adipocytes have been reported to express the main receptor for IL-8, CXCR 1, and CXCR 2 [11]. We observed that PD98059 or SB203580 inhibited the IL-8 induced IL-8 mRNA expression. Taken together, our data suggest that inflammatory stimulations may create a vicious circle of IL-8 production in human adipocytes via ERK pathway and/or p38 MAPK pathway.

In the initiation and maintenance of inflammatory reactions in the adipose tissue, IL-8 plays an important role in the recruitment of neutrophils, lymphocytes, and monocytes. However, the biological/pathological role of IL-8 expression in adipocytes and adipose tissue is not fully understood [14]. We inferred that IL-8 itself might affect insulin sensitivity in adipocytes. To test that possibility, we examined the effect of IL-8 on the insulin-induced Akt phosphorylation in fully differentiated human adipocytes. Here, we observed that the insulin-induced Akt phosphorylation was clearly diminished by IL-8; it was abrogated by PD98059 or SB203580. Harmon et al. reported that PD98059 does not affect the activation of Akt induced by insulin [15]. Hernandez et al. have suggested that ERK and p38 MAPK could be major factors in TNF-α-induced insulin resistance in brown adipocytes [16]. Those findings indicate that ERK pathway might not be necessary for insulin-induced Akt phosphorylation, but might be an important pathway for inhibition of inflammatory cytokine for insulin-induced Akt phosphorylation. Our data also suggest that IL-8 might promote insulin resistance in human differentiated adipocytes in the situation of exposure to IL-8 via the ERK pathway and/or p38 MAPK pathway. Taken together, these findings support the notion that IL-8 might have important roles in adipocyte physiology other than inflammatory cell recruitment.

In conclusion, our data suggest that IL-8 is a main adipocytokine producing insulin resistance via the inhibition of insulin-induced Akt phosphorylation in human adipocytes, and the attenuation of IL-8 production and/or action may be a target for the prevention of diabetes and its complications.

## Competing interests

The authors declare that they have no competing interests.

## Authors' contributions

CK carried out the experiments, analyzed the data and contributed in the drafting of the manuscript. SA aided in cell culture, and western blotting. MI, MI, IU, KY, KT, and MK contributed in the editing of the manuscript. MU conceived and designed of the study and supervised the project and drafted of the manuscript. All authors read and approved the final manuscript.
